# Case Report: Apremilast for Therapy-Resistant Pemphigus Vulgaris

**DOI:** 10.3389/fimmu.2020.588315

**Published:** 2020-10-27

**Authors:** Katharina Meier, Julia Holstein, Farzan Solimani, Jens Waschke, Kamran Ghoreschi

**Affiliations:** ^1^Department of Dermatology, Venereology and Allergology, Charité-Universitätsmedizin Berlin, corporate member of Freie Universität Berlin, Humboldt-Universität zu Berlin, and Berlin Institute of Health, Berlin, Germany; ^2^Department of Dermatology, University Medical Center, Eberhard Karls Universität Tübingen, Tübingen, Germany; ^3^Faculty of Medicine, Institute of Anatomy, Ludwig Maximilians University Munich, Munich, Germany

**Keywords:** pemphigus vulgaris (PV), autoimmunity, apremilast, T follicular regulatory (Tfr) cell, blistering disorders

## Abstract

**Background:**

In pemphigus, elucidating the disease-causing immune mechanism and developing new therapeutic strategies are needed. In this context, the second messenger 3′,5′-cyclic adenosine monophosphate (cAMP) is gaining attention. cAMP is important in hematological and auto-inflammatory disorders. A class of enzymes called phosphodiesterases (PDEs) control intracellular cAMP levels. In pemphigus, cAMP levels increase following IgG binding to Dsg3. This appears to be a mechanism to preserve epithelial integrity.

**Objectives:**

To determine whether apremilast, an inhibitor of the PDE4 normally used in psoriasis, may be of benefit in the blistering skin disorder pemphigus.

**Methods:**

Here we report of a 62 years old patient with chronic debilitating and recalcitrant pemphigus not responding to several previous treatments, who received treatment with apremilast over a period of 32 weeks. Desmoglein autoantibody levels were assessed by Enzyme-linked Immunosorbent Assay (ELISA), whereas disease severity and quality of life were assessed by the Autoimmune Bullous Skin Disorder Intensity Score (ABSIS). In an attempt to explain the effects of apremilast in pemphigus, peripheral blood mononuclear cells (PBMCs) were analyzed for the duration of treatment by flow cytometry for the distribution of specialized T cell subsets. The frequencies of circulating T helper (Th) 1, Th2, Th17, Th17.1 and T follicular helper (Tfh) 1, Tfh2, Tfh17, and Tfh17.1 were analyzed by CCR6, CXCR3, and CXCR5 expression of CD4^+^ T cells. Further, based on the different expressions of CXCR5, CD127, and CD25, we analyzed the T regulatory (Treg) and T follicular regulatory (Tfreg) compartment.

**Results:**

In response to apremilast treatment, Dsg-specific autoantibody titers decreased, blistering ceased and lesions healed, showing a long-lasting effect. While the frequencies of most of the Th and Tfh cell subsets remained unchanged, we observed a continuous increase in Treg and Tfreg cell levels.

**Conclusion:**

Our findings are encouraging and warrant extension of the beneficial effect of PDE4 inhibition on a larger cohort of pemphigus patients.

## Background

Pemphigus with its subtypes pemphigus vulgaris (PV) and pemphigus foliaceus (PF) is an autoimmune blistering disease that affects skin and mucosa. Autoantibodies directed against desmogleins (Dsg) are responsible for skin blistering (1). In PF, patients have autoantibodies against Dsg1, whereas in PV, circulating autoantibodies against both Dsg3 and Dsg1 are present. Dsg are constitutive parts of desmosomes, protein complexes responsible for keratinocyte adhesion. Binding of Dsg by autoantibodies results in loss of cell adhesion and acantholysis (1). PV is clinically characterized by oral and cutaneous erosions, ranging from fragile cutaneous bullae to strongly debilitating erosions of the nasopharyngeal region (1). Treatment guidelines recommend systemic corticosteroids in combination with immunosuppressive agents such as azathioprine or mycophenolate mofetil. A recently approved therapeutic for pemphigus is the CD20-targeting monoclonal antibody rituximab, which depletes pre-B- and mature B-lymphocytes. Other treatments such as methotrexate, cyclophosphamide, dapsone, intravenous immunoglobulins or immunoapheresis are third line options (2). The aforementioned treatments have improved the prognosis, but adverse effects and complications especially from long-term immunosuppressive therapies still contribute to morbidity and mortality. Unraveling the pathogenic mechanisms helps to identify new therapeutic targets in pemphigus. Examples are inhibitors of Bruton’s kinase (NCT03762265) or the development of a chimeric T cell antigen receptor targeting autoreactive B cells (3, 4). Phosphodiesterases (PDEs) are a family of enzymes (PDE1 to 11), which are capable of degrading cAMP intracellularly (5). In the setting of inflammatory skin diseases, PDE4 seems to be the most relevant subtype since it is widely expressed in the immune, endothelial, and epithelial cells (5). Binding of IgG to Dsg3 is a cardinal point for disease onset in pemphigus. This process causes steric hindrance and most importantly, activation of different signaling pathways. IgG binding to Dsg3 activates p38MAPK, Src, and cAMP signaling pathways (6, 7), whereas, in the cutaneous restricted PF, binding of IgG to Dsg1 exerts its pathogenic effect in in an Erk-, p38MAPK- and PKC-dependent fashion (7). As reported in experimental models, interference with these pathways can prevent development of disease (6). According to these experimental findings, here we report that the PDE4 inhibitor (PDE4i) apremilast results in cessation of blistering in PV in humans.

## Case Presentation

**Figure 1:**
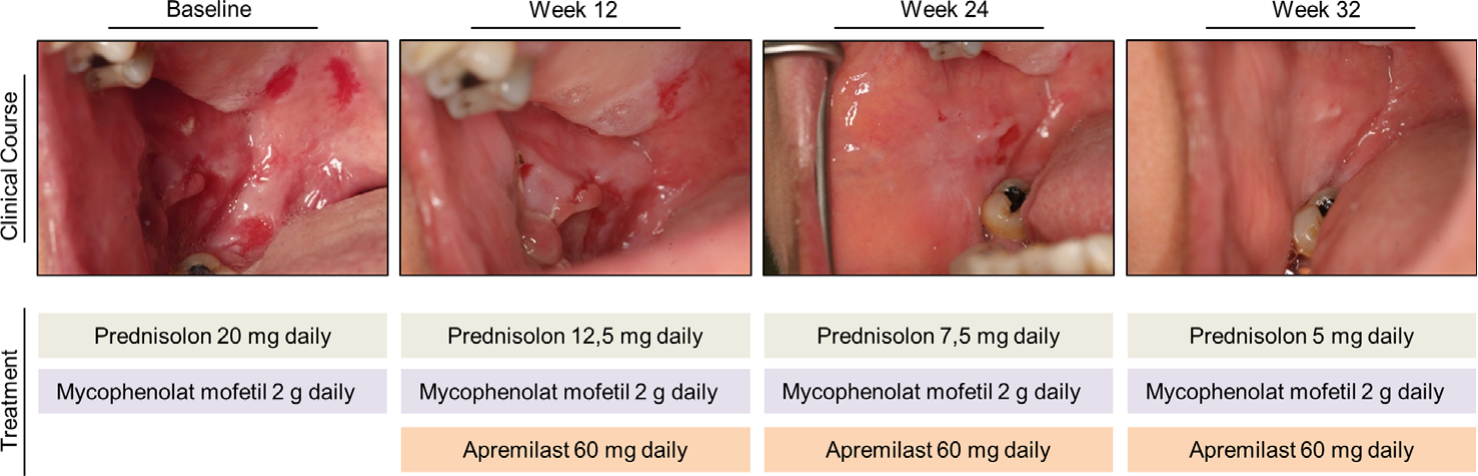
Clinical presentation of the patient’s oral cavity at baseline, at weeks 12, 24, and 32 during apremilast treatment. The medication regimen at baseline and at indicated time points is shown.

Here we report the case of a 62-year-old woman with chronic, non-responsive PV, successfully treated over a period of 32 weeks with apremilast 30 mg twice daily. PV diagnosis was made in 2015 by clinical appearance and further confirmed by laboratory diagnostics. The patient presented with extensive painful erosions of the oral cavity (**Figure 1**). At the time of the initial consultation, histology showed suprabasal loss of epidermal adhesion and acantholysis. Direct immunofluorescence demonstrated typical intercellular IgG deposition within the epidermis. Enzyme-linked immunosorbent assays (ELISAs) were positive for anti‐Dsg1 and anti‐Dsg3 autoantibodies. Previous therapies adopted over the years with azathioprine, mycophenolate mofetil, dapsone, and intravenous immunoglobulins had failed. Repeated attempts to reduce the dose of systemic steroids below 20 to 30 mg prednisone equivalent were not successful, and tapering was linked to the reappearance of painful and severe oral erosions. Lastly, a therapy with rituximab 2× 1,000 mg in combination with mycophenolat mofetil and prednisone was of no clinical benefit. Due to the exhaustion of existing treatment options and based on preclinical data from *in vitro* and *in vivo* models suggesting that PDE4 inhibition may help to control autoantibody-mediated disorders (7–9) apremilast 30 mg twice a day (standard psoriasis regimen) was initiated as add-on therapy to 20 mg prednisone and 2 g mycophenolat mofetil background treatment. Informed consent was obtained for compassionate use.

## Methods

### Detection of Anti-Desmoglein Autoantibodies

The presence of IgG autoantibodies against Dsg1 or Dsg3 in patient’s sera was evaluated by anti-Dsg1- and anti-Dsg3-ELISA according to the manufacturer’s protocol (Euroimmun, Lübeck, Germany).

### Flow Cytometric Analysis

Peripheral blood mononuclear cells (PBMCs) were isolated from citrate-phosphate-dextrose-adenine (CPDA)-containing peripheral blood samples by density gradient centrifugation using lymphocyte separation medium (Capricorn Scientific, Ebsdorfergrund, Germany) and stained for flow cytometry with the following monoclonal antibodies:CXCR3-BV421 (G025H7), CD4-BV510 (RPA-T4), CD45RA-FITC (HI100), CD3-PerCP-Cy5.5 (SK7), CXCR5-PE (J252D4), CCR6-APC (G034E3), CD127-PE-Dazzle/Texas Red (A019D5), CD25-PE-Cy7 (M-A251), CD19-FITC (HI), CD24-PerCP-Cy5.5 (ML5), CD27-PE-Cy7 (M-T271), CD38-BV650 (HB-7). Cells were analyzed using BD FACS LSR II (BD Biosciences, San Jose, USA).

### Laboratory Investigations

**Figure 2:**
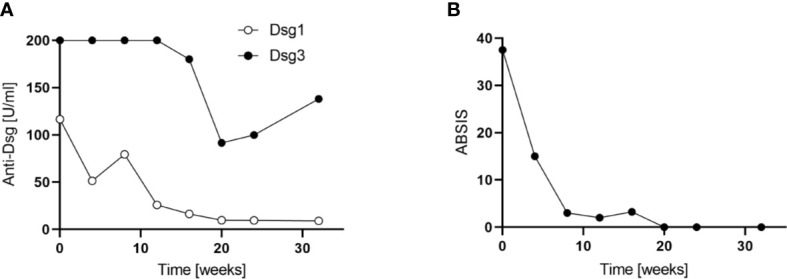
**(A)** Anti-desmoglein (Dsg)1 and -Dsg3 autoantibody levels during apremilast treatment (32 weeks). Serum autoantibodies were determined by Enzyme-linked Immunosorbent Assays ELISA (Euroimmune, Lübeck, Germany). **(B)** Clinical efficacy of apremilast treatment as assessed by the Autoimmune Bullous Skin Disorder Intensity Score (ABSIS).

**Figure 3:**
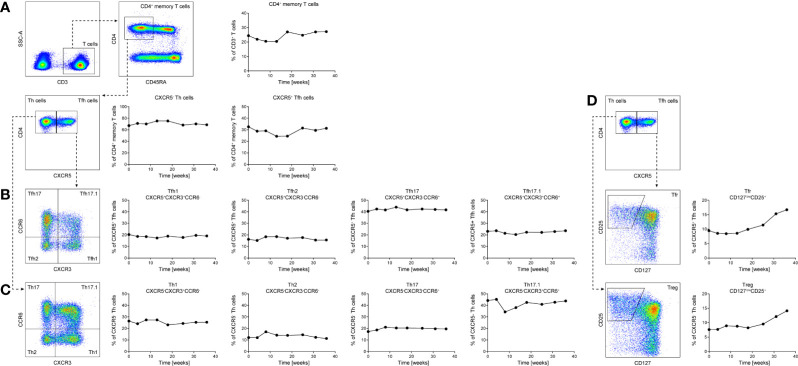
Longitudinal assessment of the patient’s circulating T follicular helper (Tfh) **(A, B)**, T helper **(A, C)** and T regulatory (Treg)/T follicular regulatory (Tfreg) cells **(A, D)** subsets over a 32-week period of apremilast treatment. CD45RA^−^CD4^+^ memory T cells were analyzed for their surface expression of CXCR5, CCR6, and CXCR3 by flow cytometry **(A–C)**. Levels of Treg/Tfreg cells were determined by cell surface staining for CXCR5, CD25, and CD127 of CD45RA^–^CD4^+^ memory T cells **(A, D)**. Peripheral blood was stained with antibodies directed against the indicated cell surface receptors **(A–D)** and analyzed by flow cytometry (BD LSR II and flowjo Software).

Treatment with apremilast led to a rapid and meaningful decrease in disease activity as determined by a reduction of the Autoimmune Bullous Skin Disorder Intensity Score (ABSIS) score from 38 to 0 and a suppression of anti-Dsg1 and anti-Dsg3 autoantibody levels in serum (**Figures 2A, B**). Concomitantly, the patient showed an improvement in quality of life. The clinical improvement due to apremilast treatment allowed for reduction of the prednisone dose below 20 mg for the first time, and finally, a reduction to 5 mg daily maintenance dose was tolerated without worsening of symptoms (**Figure 1**). During treatment with apremilast we performed an immunological monitoring of the effects on peripheral circulating T cells by cell surface staining and flow cytometric analysis as reported elsewhere (10). Based on the surface expression of chemokine receptors we followed the levels of T helper (Th) and T follicular helper (Tfh) cell subsets with type 1, 2, 17, and 17.1 profiles in the circulation (**Figures 3A–C**) (10). Additionally, we monitored levels of circulating T regulatory (Treg) and T follicular regulatory (Tfreg) cells (**Figure 3D**). At baseline, we detected dominant Th17 (40% of total Th cells) and Tfh17.1 (45% of total Tfh cells) cell subsets (**Figures 3B, C**). However, we did not observe meaningful changes in the distribution of type 1, 2, 17 or 17.1 Th or Tfh cell subsets over time. Remarkably, we found an increase in circulating Treg and Tfreg cell subsets throughout treatment with apremilast. The proportion of Treg and Tfreg cells increased continuously during the 30-week treatment period (from ~9% to ~17% and 7.5% to 15%, respectively) (**Figure 3D**).

## Discussion

Initially introduced in the setting of neoplastic malignancies (11), inhibition of PDE4 appears to be a successful way of managing several inflammatory skin diseases. Though approved only for plaque psoriasis, psoriatic arthritis and Behcet’s disease, off-label reports with limited patient numbers suggest efficacy in discoid lupus erythematosus, atopic dermatitis, pytiriasis rubra pilaris, and generalized granuloma anulare. PDE4i seems to be effective in the management of laminin *γ*-1 pemphigoid as reported in an individual with concomitant psoriasis and in a preclinical model of epidermolysis bullosa acquisita (8, 12). Both are blistering disorders with skin fragility, driven by autoantibodies against laminin *γ*-1 and collagen VII, respectively. *In vitro*, co-culture of keratinocytes with Dsg3 autoantibodies led to an increase of cAMP levels and impaired p38MAPK activation. Interfering with intracellular cAMP levels may be a strategy to protect epidermal integrity (9). In addition to effects on epithelial cells, apremilast may exert immunomodulatory effects. The accumulation of cAMP by PDE4 inhibition activates protein kinase A, and this decreases the expression of pro-inflammatory mediators such as IL-17 or IFN-*γ* (13, 14). Furthermore, PDE4i promotes regulatory T cells and regulatory IL-10-producing B cells (15, 16). In line with this, our flow cytometry analysis of peripheral T cells demonstrated a consistent increase in Treg and Tfreg cells during PDE4 inhibition, while autoantibody levels decreased (**Figure 3D**). Since Tfh cells promote B cell mediated autoantibody production, a mechanism that seems to be relevant in pemphigus (17, 18), increasing Treg/Tfreg numbers may exert an inhibitory effect on Tfh and B cell activity (19). On the contrary, we could not observe an effect of apremilast on the Th and Tfh inflammatory subsets, suggesting that dampening of the inflammatory process may be due to overactivation of the regulatory compartment.

Taken together, our findings on a patient with pemphigus treated with the PDE4i apremilast as add-on therapy to background mycophenolat mofetil 2 g daily and prednisolone 5 mg, shows that PDE4 inhibition is a safe and effective novel treatment option. Meaningful clinical improvement and suppression of autoantibodies were achieved 12 weeks after PDE4i treatment was initiated. The clinical and serological response was accompanied by an increase of peripheral Treg and Tfreg cells over a treatment period of 30 weeks. Future studies are needed to establish apremilast as an add-on small molecular therapeutic in treatment-resistant pemphigus.

## Data Availability Statement

The raw data supporting the conclusions of this article will be made available by the authors, without undue reservation.

## Ethics Statement

Ethical review and approval was not required for the study on human participants in accordance with the local legislation and institutional requirements. The patients/participants provided their written informed consent to participate in this study. Written informed consent was obtained from the individuals for the publication of any potentially identifiable images or data included in this article.

## Author Contributions

KM, FS JH, JW, and KG designed the manuscript, performed the experiment, and designed figures. KG supervised the study and revised critically the final version of the manuscript. All authors contributed to the article and approved the submitted version.

## Funding

The study was funded by grants from the German Research Foundation (Deutsche Forschungsgemeinschaft, DFG); FOR 2497/TP02 (GH133/2-1) to KG.

## Conflict of Interest

KG and KM have received honoraria or travel expenses for lecture and research activities from Celgene.

The remaining authors declare that the research was conducted in the absence of any commercial or financial relationships that could be construed as a potential conflict of interest.

## References

[1] Kasperkiewicz M Ellebrecht CT Takahashi H Yamagami J Zillikens D Payne AS Pemphigus. Nat Rev Dis Primers (2017) 3:17026. 10.1038/nrdp.2017.26 28492232 PMC5901732

[2] Amber KT Maglie R Solimani F Eming R Hertl M Targeted Therapies for Autoimmune Bullous Diseases: Current Status. Drugs (2018) 78:1527–48. 10.1007/s40265-018-0976-5 30238396

[3] Ellebrecht CT Bhoj VG Nace A Choi EJ Mao X Cho MJ Reengineering chimeric antigen receptor T cells for targeted therapy of autoimmune disease. Science (2016) 353:179–84. 10.1126/science.aaf6756 PMC5343513 27365313

[4] Didona D Maglie R Eming R Hertl M Pemphigus: Current and Future Therapeutic Strategies. Front Immunol (2019) 10:1418. 10.3389/fimmu.2019.01418 31293582 PMC6603181

[5] Jeon YH Heo YS Kim CM Hyun YL Lee TG Ro S Phosphodiesterase: overview of protein structures, potential therapeutic applications and recent progress in drug development. Cell Mol Life Sci (2005) 62:1198–220. 10.1007/s00018-005-4533-5 PMC11139162 15798894

[6] Spindler V Eming R Schmidt E Amagai M Grando S Jonkman MF Mechanisms Causing Loss of Keratinocyte Cohesion in Pemphigus. J Invest Dermatol (2018) 138:32–7. 10.1016/j.jid.2017.06.022 29037765

[7] Walter E Vielmuth F Rotkopf L Sardy M Horvath ON Goebeler M Different signaling patterns contribute to loss of keratinocyte cohesion dependent on autoantibody profile in pemphigus. Sci Rep (2017) 7:3579. 10.1038/s41598-017-03697-7 28620161 PMC5472593

[8] Koga H Recke A Vidarsson G Pas HH Jonkman MF Hashimoto T PDE4 Inhibition as Potential Treatment of Epidermolysis Bullosa Acquisita. J Invest Dermatol (2016) 136:2211–20. 10.1016/j.jid.2016.06.619 27388992

[9] Spindler V Vielmuth F Schmidt E Rubenstein DS Waschke J Protective endogenous cyclic adenosine 5’-monophosphate signaling triggered by pemphigus autoantibodies. J Immunol (2010) 185:6831–8. 10.4049/jimmunol.1002675 PMC3129745 21037102

[10] Morita R Schmitt N Bentebibel SE Ranganathan R Bourdery L Zurawski G Human blood CXCR5(+)CD4(+) T cells are counterparts of T follicular cells and contain specific subsets that differentially support antibody secretion. Immunity (2011) 34:108–21. 10.1016/j.immuni.2010.12.012 PMC3046815 21215658

[11] Cooney JD Aguiar RC Phosphodiesterase 4 inhibitors have wide-ranging activity in B-cell malignancies. Blood (2016) 128:2886–90. 10.1182/blood-2016-09-737676 PMC5179339 27756749

[12] Waki Y Kamiya K Komine M Maekawa T Murata S Ishii N A case of anti-laminin gamma1 (p200) pemphigoid with psoriasis vulgaris successfully treated with apremilast. Eur J Dermatol (2018) 28:413–4. 10.1684/ejd.2018.3280 29619999

[13] Chen W Wang J Xu Z Huang F Qian W Ma J Apremilast Ameliorates Experimental Arthritis via Suppression of Th1 and Th17 Cells and Enhancement of CD4(+)Foxp3(+) Regulatory T Cells Differentiation. Front Immunol (2018) 9:1662. 10.3389/fimmu.2018.01662 30072998 PMC6058600

[14] McCann FE Palfreeman AC Andrews M Perocheau DP Inglis JJ Schafer P Apremilast, a novel PDE4 inhibitor, inhibits spontaneous production of tumour necrosis factor-alpha from human rheumatoid synovial cells and ameliorates experimental arthritis. Arthritis Res Ther (2010) 12:R107. 10.1186/ar3041 20525198 PMC2911898

[15] Bopp T Dehzad N Reuter S Klein M Ullrich N Stassen M Inhibition of cAMP degradation improves regulatory T cell-mediated suppression. J Immunol (2009) 182:4017–24. 10.4049/jimmunol.0803310 19299699

[16] Mavropoulos A Zafiriou E Simopoulou T Brotis AG Liaskos C Roussaki-Schulze A Apremilast increases IL-10-producing regulatory B cells and decreases proinflammatory T cells and innate cells in psoriatic arthritis and psoriasis. Rheumatol (Oxford) (2019) 58:2240–50. 10.1093/rheumatology/kez204 31209492

[17] Hennerici T Pollmann R Schmidt T Seipelt M Tackenberg B Mobs C Increased Frequency of T Follicular Helper Cells and Elevated Interleukin-27 Plasma Levels in Patients with Pemphigus. PloS One (2016) 11:e0148919. 10.1371/journal.pone.0148919 26872212 PMC4752242

[18] Kim AR Han D Choi JY Seok J Kim SE Seo SH Targeting inducible costimulator expressed on CXCR5(+)PD-1(+) TH cells suppresses the progression of pemphigus vulgaris. J Allergy Clin Immunol (2020) 18:S0091-6749(20)30493-0. 10.1016/j.jaci.2020.03.036 32311391

[19] Sage PT Ron-Harel N Juneja VR Sen DR Maleri S Sungnak W Suppression by TFR cells leads to durable and selective inhibition of B cell effector function. Nat Immunol (2016) 17:1436–46. 10.1038/ni.3578 PMC5502675 27695002

